# Relationship of diagnostic accuracy of renal cortical echogenicity with renal histopathology in dogs and cats, a quantitative study

**DOI:** 10.1186/s12917-016-0941-z

**Published:** 2017-01-17

**Authors:** Tommaso Banzato, Federico Bonsembiante, Luca Aresu, Alessandro Zotti

**Affiliations:** 1 Department of Animal Medicine, Production and Health, Clinical Section, Radiology Unit, University of Padua, Viale dell’Università 16, Legnaro, 35020 Padua Italy; 2 Department of Comparative Biomedicine and Food Science, University of Padua, Viale dell’Università 16, Legnaro, 35020 Padua Italy

**Keywords:** Kidney, Ultrasound, Image analysis, Pathology

## Abstract

**Background:**

Renal cortical echogenicity is routinely evaluated during ultrasonographic investigation of the kidneys. Both in dog and cat previous *ex-vivo* studies have revealed a poor correlation between renal echogenicity and corresponding lesions. The aim of this study was to establish the in-vivo relationship between renal cortical echogenicity and renal histopathology.

**Results:**

Thirty-eight dogs and fifteen cats euthanized for critical medical conditions were included in the study. Ultrasonographic images of both kidneys were acquired *ante mortem* at standardized ultrasonographic settings. The echogenicity was quantified by means of Mean Gray Value (MGV) of the renal cortex measured with ImageJ. A complete histopathological examination of both kidneys was performed. Five kidneys were excluded because histopathology revealed neoplastic lesions. Only samples affected by tubular atrophy showed statistically different values in dog, and histopathology explained 13% of the total variance. MGV was not correlated neither to the degeneration nor to the inflammation scores. However, significant differences were identified between mildly and severely degenerated samples. Overall, the classification efficiency of MGV to detect renal lesions was poor with a sensitivity of 39% and a specificity of 86%.

In cats, samples affected by both tubular vacuolar degeneration and interstitial nephritis were statistically different and histopathology explained 44% of the total variance. A linear correlation was evident between degeneration and MGV, whereas no correlation with inflammation was found. Statistically significant differences were evident only between normal and severely degenerated samples with a sensitivity of 54.17% and a specificity of 83.3% and MGV resulted scarce to discriminate renal lesions in this species.

**Conclusions:**

Renal cortical echogenicity shows low relevance in detecting chronic renal disease in dog whereas it results worth to identify severe renal damage in cat.

## Background

A thorough ultrasonographic inspection of the kidneys is mandatory in the clinical examination of dogs and cats with suspected or confirmed renal disease. The shape, size, contours, renal pelvis, renal medulla, corticomedullary distinction and renal cortical echogenicity are routinely evaluated [1, 2]. Focal renal diseases such as tumors, cysts or hematomas are readily detected using B-Mode ultrasound [1, 2], whereas the evaluation of diseases involving the entire kidney are more challenging since the relative ultrasonographic changes are usually subtle and non-specific. Increased renal cortical echogenicity was previously reported to be a common finding associated with renal insufficiency [1]. However, animals affected by severe renal insufficiency may show normal kidneys during ultrasonography [2].

To date, no in-vivo studies correlating renal cortical echogenicity and diffuse renal pathology in dogs and cats have been published. The relationship between renal cortical echogenicity and renal pathology was previously studied by the authors in a standardized *ex-vivo* study [3].

The aims of this present study are: 1) to describe in a standardized in-vivo operative conditions the relationship between renal cortical echogenicity and renal histopathology in dogs and cats; 2) to determine whether renal cortical echogenicity could be used to discriminate diffuse renal pathology.

## Methods

### Animals

All the animals included in this study were admitted for medical consult at the Veterinary Teaching Hospital, University of Padua, Italy, from January 2015 to May 2016. Ultrasonography was part of the routine clinical evaluation and was performed within 24 h prior to death. Written consent was obtained from the owners and a complete clinical history was obtained. All the animals were euthanized due to critical medical conditions unrelated to the purposes of the study.

A full necropsy was performed within 3 h from death on every cadaver and both kidneys were collected and formalin fixed.

### Ultrasonographic procedures

All the ultrasounds were performed with a 4- to 9 MHz micro convex array transducer connected to a commercial sonographic scanner (Zonare, Zonare Medical Systems Inc, Mountain View, California, USA). The following ultrasonographic settings were selected in all the scans: abdominal preset, depth 4 cm, frequency 8.5 MHz, gain 90; time-gain-compensation control settings were maintained in a neutral position. Longitudinal and transversal scans were obtained for each kidney. Each scan included both the cortex and medulla.

### Image analysis procedures

Images were stored in a digital imaging and communications in medicine format (DICOM) without compression. Mean Gray Value (MGV) calculated with an open-source sofware (ImageJ, 1.480 version, National Institutes of Health, Bethesda, USA) was used to quantify the echogenicity in three square 64x64 pixel region of interest (RoI) and then averaged. MGV is the sum of the pixel intensities within a given RoI divided by the number of pixels included in the RoI assuming a eight bit image with 256 possible shades of gray (2^8^ combinations in binary code). The RoIs were always placed at about 1-cm depth within the cortical side proximal to the probe immediately under each kidney serosa. Care was taken to avoid focal lesions such as mineralizations or cysts.

### Histological analysis

The kidney samples for histological analysis were processed routinely and paraffin embedded. Both right and left kidney were analyzed for each animal; serial 3 μm sections were obtained and stained routinely with specific renal stainings [3]. A set of morphological lesions was selected for the study including glomerulosclerosis, interstitial nephritis, glomerulonephritis, tubular atrophy, tubular necrosis, fibrosis, glomerular lipidosis, tubular vacuolar degeneration, amyloidosis. Additionally, the presence or absence of proximal tubular lipidosis was recorded in cat. Each kidney was considered as an independent sample. The samples revealing “no relevant findings” were considered as normal. Tumors were excluded from the study. All the histopathological lesions were recorded as present or absent and reported as a dichothomous variable. Furthermore, a 0 to 3 (0 = none, 1 = mild, 2 = moderate and 3 = severe) semi-quantitative degeneration (including glomerulosclerosis, tubular necrosis, fibrosis, glomerular and interstitial lipidosis, tubular atrophy, amyloidosis) and inflammation (including interstitial nephritis and glomerulonephritis) scores were assigned. The samples were analyzed in a blinded fashion by two veterinary pathologists (LA and FB), and differences in scores were resolved by consensus achieved through simultaneous viewing using a dual-headed microscope.

### Statistical analysis

A commercially available software was used to perform the statistical analysis (IBM Corp. Released 2011. IBM SPSS Statistics for Windows, Version 20.0. Armonk, NY: IBM Corp.). The dog and cat data were analyzed separately. The variable MGV was tested for normality using the Shapiro-Wilk test. The MGV calculated on each kidney was included in the statistical analysis. The presence or absence of individual histopathological lesions was considered as a dichotomous variable and differences between groups were tested between samples showing a specific lesion and all the other samples. Analysis of variance (ANOVA) was used to calculate differences between groups for normally distributed data. Differences between groups in non-normally distributed data were investigated with the Mann-Whitney U test. A univariate analysis was performed to evaluate the variance of MGV explained by histological parameters.

Correlation between MGV and degeneration and inflammation score was tested by means of the Spearman rank-order test. Differences between inflammation and degeneration score groups in normally distributed data were calculated using ANOVA. Kruskal-Wallis H test was used to study differences between groups in non-normally distributed data. Multiple comparison tests were performed with the Tukey-Kramer method to test which of both the degeneration and inflammation score groups were significantly different from the others. The diagnostic performance of MGV in the detection of pathological samples (samples that were not classified as “no relevant findings”) was tested by means of the receiver operating characteristic curve (ROC). In all the statistical analysis a *P* < 0.05 was considered significant.

## Results

### Dogs

Thirty-eight adult dogs (13 females and 25 males) weighing from 3 to 45 kg (mean age: 9.5 ± 4 years) were recruited for the study. Descriptive statistics of MGV in the dog samples classified by histopathological lesion are reported in Table 1. Twenty-one kidneys were classified as normal by optical microscopy examination. Five kidneys were classified as neoplastic and were excluded from further analyses. MGV resulted from the Shapiro-Wilk test as not normally distributed and therefore differences between groups were tested by means of the Mann-Whitney U test. Only the MGV calculated on samples affected by tubular atrophy resulted as significantly different (U = 470, z = 2.74, *p* = 0.006) between groups. The univariate analysis was performed with tubular atrophy as independent variable and MGV as dependent variable. The assumptions of linearity, independence of errors, homoscedasticity, unusual points and normality of residuals were met. Tubular atrophy accounted 13.4% of total MGV variability (F(2–66) = 11.18, *P* = 0.001, adj. *R* ^2^ = 0.13).

**Table 1:** Descriptive statistics and results of the Mann-Whitney U test of MGV calculated on the dog samples classified on the basis of histopathological lesions

		Presence of the considered lesion			Absence of the considered lesion		
Histological changes	No. of samples	Mean ± SD	Median	Range	Mean ± SD	Median	Range
No relevant findings	21	49.88 ± 18.7	41.24	26.9–93.8	47.96 ± 21.42	38.76	16.16–94.76
Glomerulosclerosis	33	47.79 ± 22.88	37.7	16.1–94.7	48,68 ± 18.67	41.36	25.47–93.08
Tubular atrophy*	11	66.8 ± 21.78	66.94	31.42–94.76	44.93 ± 18.63	37.77	16.13–93.08
Fibrosis	12	51.83 ± 27.68	44.54	20.23–94.76	47.53 ± 19.04	40.07	16.16–93.08
Glomerular atrophy	10	48.16 ± 20.94	41.16	25.69–77.35	48.29 ± 20.76	40.91	16.16–94.76
Interstitial nephritis	23	48.94 ± 22.61	44.03	20.23–94.76	47.96 ± 19.76	39.54	16.16–93.08
Glomerulonephritis	9	37.34 ± 8.75	35.52	25.69–49.36	49.83 ± 21.31	41.63	16.16–94.76
Neoplasia	5	33.05 ± 6.7	33.69	25.47–41.63	49.20 ± 20.81	44.03	16.16–94.76

*Statistically significant differences –*p* < 0,05

**Fig. 1:**
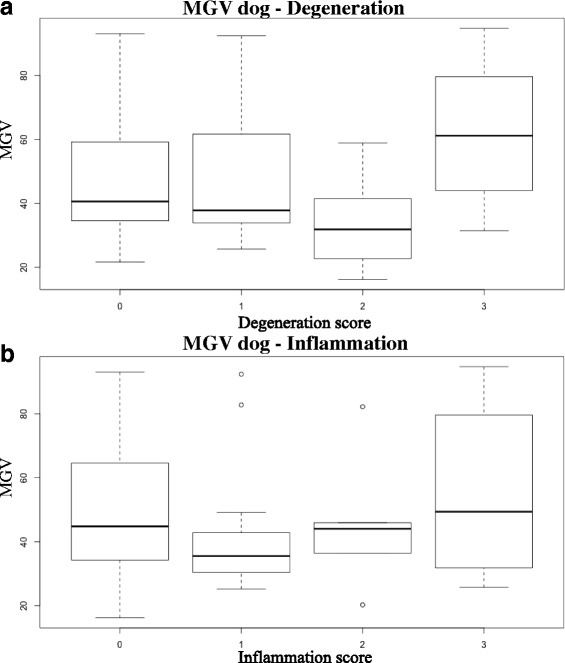
Box-and-whisker plots of MGV in the dog samples classified on the basis of the degeneration (**a**) and inflammation (**b**) scores assigned by the pathologists

Descriptive statistics (Table 2) and box-and-whisker plots (Fig. 1) of MGV, classified on the basis of the degeneration and inflammation scores assigned by the pathologists, are reported. As a result of the Spearman rank-order correlation test there was no significant association between MGV and degeneration and inflammation scores. However, degeneration scores showed significant differences by Kruskal-Wallis H test (χ2 = 8.55; *p* = 0.036), more specifically post hoc test revealed significant differences between the grade 2 and grade 3 of the degeneration (Fig. 1). No significant results were evident for the inflammation score.

**Table 2:** Descriptive statistics of MGV of the dog samples classified on the basis of the degeneration and inflammation scores assigned by the pathologists

	Degeneration				Inflammation			
Score	No. of samples	Mean ± SD	Median	Range	No of samples	Mean ± SD	Median	Range
0	26	48.30 ± 19.25	40.91	21.61–93.08	44	51.1 ± 19.5	49.4	16.16–93.08
1	20	49.23 ± 21.32	38.26	25.69–92.41	13	42.45 ± 21.18	33.86	25.17–92.41
2	7	33.35 ± 15.5	30.30	16.16–58.57	5	45.76 ± 22.75	44.03	20.23–82.21
3	14	59.75 ± 21.21	61.47	31.42–94.76	5	56.25 ± 30.03	49.36	25.69–94.76

**Fig. 2:**
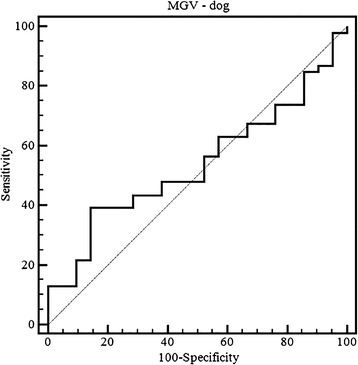
ROC curve analysis of the dog samples classified as normal (“no relevant findings”) and as pathological. (AUROC = 0.54; 95% confidence interval = 0.32–0.60). Choosing a cut-off value of 35 sensitivity was 39% and specificity was 86%)

Finally, the ROC curve analysis showed a poor diagnostic performance of MGV (area under the receiver characteristics curve (AUROC) = 0.54; 95% confidence interval 0.32–0.60). Using a cut-off value of 35, sensitivity was 39% and specificity was 86% (Fig. 2).

### Cats

Fifteen adult cats (five females and ten males) weighing from 3 to 9 kg (mean age 9.3 ± 4.1 years) were involved in the study. The descriptive statistics of MGV for cat are reported in Table 3. Six samples were classified as normal. A non-normal distribution of MGV was shown by Shapiro-Wilk test and therefore differences between groups were calculated by means of the Mann-Whitney U test. Differences between groups were evident for fibrosis (U = 56, z = 2.33, *p* = 0.002), tubular vacuolar degeneration (U = 195, z = 6.43, *p* = 0.000), interstitial nephritis (U = 117, z = 3.04, *p* = 0.002), and glomerulonephritis (U = 0.5, z = −2.21, *p* = 0.005). Since only two samples showed fibrosis and glomerulonephritis, these latter histological lesions were not considered in further analysis. Only histopathological lesions showing statistically significant differences were included in the univariate analysis. The assumptions of linearity, independence of errors, homoscedasticity, unusual points and normality of residuals were met. Both tubular vacuolar degeneration and interstitial nephritis were included in the model, F(2–29) = 15.31, *P* < 0.000, adj. *R* ^2^ = 0.44. Results of the univariate analysis are reported in Table 4.

**Table 3:** Descriptive statistics and results of the Mann-Whitney U test of MGV calculated on the cat samples classified on the basis of histopathological lesions

		Presence of the considered lesion			Absence of the considered lesion		
Histological changes	No. of samples	Mean ± SD	Median	Range	Mean ± SD	Median	Range
No relevant findings	6	36 ± 15.73	33.15	20.04–62.54	46.77 ± 22.05	46.43	17.86–110.97
Glomerulosclerosis	10	51.41 ± 32.67	49.92	17.86–110.97	41.23 ± 11.8	43.25	17.86–110.97
Proximal tubular lipidosis	24	47.79 ± 22,76	46.67	17.76–110.97	34.16 ± 10.57	33.83	22.94–48.57
Tubular atrophy	5	34.75 ± 7.87	34.19	24.5–46.67	46.6 ± 22.46	46.18	17.86–110.97
Fibrosis*	2	104.9 ± 8.52	104.9	98.84–110.97	40.32 ± 13.72	43.25	17.86–62.54
Tubular vacuolar *degeneration	15	56.18 ± 21.93	51.27	23.64–110.97	33.06 ± 12.54	34.06	17.86–62.54
Glomerular atrophy	9	41.6 ± 10.18	43.61	22.94–54.8	45.92 ± 24.5	44.13	17.86–110.97
Interstitial nephritis*	5	75.77 ± 27.24	60.01	49.67–110.97	38.39 ± 13.15	37.57	17.86–62.54
Glomerulonephritis*	2	18.76 ± 1.2	18.76	17.86–19.67	46.47 ± 20.64	45.15	20.05–110.97

*Statistically significant differences –*p* < 0,05

**Table 4:** Results of univariate analysis of the cat samples with MGV as dependent variable

Histological changes	B	Standard error	Beta
Constant	33.65	3.86	
Tubular vacuolar degeneration	13.329	6.11	0.321*
Interstitial nephritis	29.238	8.2	0.527*

B: unstandardized regression coefficient, Beta: standardized coefficient, *P value < 0.05

**Fig. 3:**
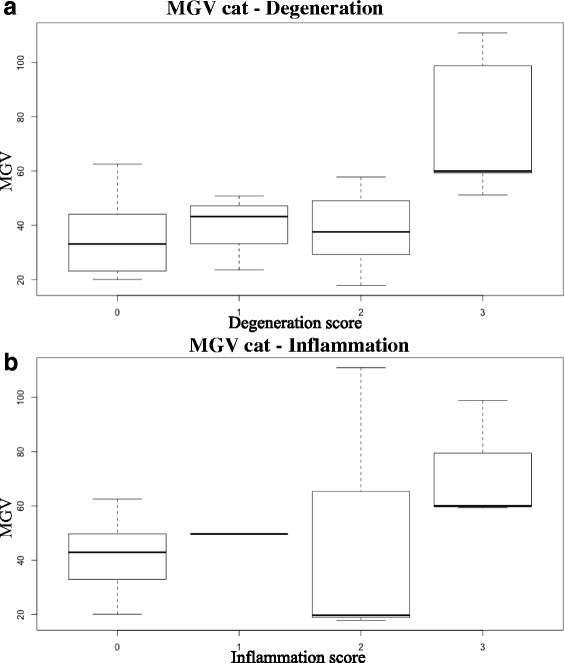
Box-and-whisker plots of MGV in the cat samples classified on the basis of the degeneration (**a**) and inflammation (**b**) scores assigned by the pathologists

Descriptive statistics and box-and-whisker plots (Fig. 3) for degeneration and inflammation are reported in Table 5. As a result of the Spearman rank-order correlation test, MGV and degeneration scores were significantly correlated (*r* = 0.44, *p* = 0.014), whereas MGV and inflammation scores were not correlated. Significant differences, calculated by means of the Kruskal-Wallis H test, were evident between degeneration score groups (χ2 = 10.05; *p* = 0.018); post-hoc test revealed significant differences between grade 0 and 3 and between grade 2 and 3 degeneration score groups (Fig. 1). No significant differences were evident between the inflammation score groups.

**Table 5:** Descriptive statistics of MGV of the cat samples classified on the basis of the degeneration and inflammation scores assigned by the pathologists

	Degeneration				Inflammation			
Score	No. of samples	Mean ± SD	Median	Range	No. of samples	Mean ± SD	Median	Range
0	6	36.03 ± 15.5	33.15	20.04–62.54	23	40.1 ± 12.27	42.88	20.04–62.54
1	4	40.25 ± 11.66	43.25	23.61–50.89	1	-	-	-
2	15	38.73 ± 13.23	37.57	17.86–57.81	3	49.5 ± 52.24	19.67	17.86–110.97
3	5	76.09 ± 26.87	60.01	51.26–110.97	3	72.74 ± 22.6	60.01	59.36–98.84

**Fig. 4:**
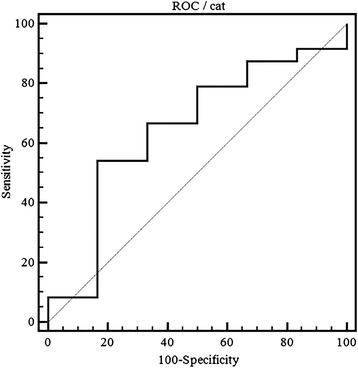
ROC curve analysis of the cat samples classified as normal (“no relevant findings”) and as pathological. (AUROC = 0.65, 95%; confidence interval:= 0.45–0.81). Choosing a cut-off value of 44 the sensitivity was 54% and the specificity was 83%)

ROC curve analysis revealed that MGV had poor discriminating power between normal and pathological samples (AUROC = 0.65, 95% confidence interval 0.45–0.81). Choosing a cut-off value of 44% the sensitivity was 54% and specificity was 83% (Fig. 4).

## Discussion

All previous in-vivo veterinary studies reported in the literature to objectively evaluate renal cortical echogenicity were performed in healthy animals [4–6]. These papers presented renal cortical echogenicity assessed by means of a tissue equivalent phantom [4] or in comparison to other organs [5, 6]. It is the authors’ opinion that both these approaches have some limitations; comparison with a tissue equivalent phantom is a complicated and time consuming procedure [4]. On the other hand, comparison with other organs is unreliable because: 1) renal cortical echogenicity in dogs with normal renal function is reported to be hypo, iso- or hyper-echoic to the liver [5]; 2) it assumes the spleen or the liver to be normal.

In the present work, the authors have identified the use of constant ultrasonographic settings (frequency, gain, time gain compensation, etc.) and sampling procedures to obtain comparable results. Interestingly, the variance expressed by the model was similar to a previous *ex-vivo* study [3] in dogs (12% in the *ex-vivo* study and 13% in the present in-vivo study) whereas it resulted higher in cats (23% in the *ex-vivo* study and 44% in the present in-vivo study). In our opinion, this might indicate that the patient related factors (such as body size, nutritional status) only actually accounts for a marginal part of the variability in kidney echogenicity. However, the diagnostic accuracy of MGV in the detection of pathological kidneys was lower in this in-vivo report than in our previous *ex-vivo* study, both for dogs and cats [3]. It is authors’ opinion that the above differences might be, at least partially, explained by the smaller sample size of the present in-vivo study compared to that of the previous *ex-vivo* research [3].

The relationship between the MGV calculated in the dog samples and the degeneration score assigned by the pathologists was nonlinear, and statistically significant differences were evident only between grade 2 and 3 degeneration. Moreover, the mean MGV of the kidney samples graded as two on our degeneration score scale was lower than that of the remaining degeneration score groups. A similar relationship between MGV and degeneration was evident in the *ex-vivo* report [3].

ROC curve analysis calculated in order to detect pathological kidney changes shows a very low diagnostic accuracy of MGV. This is a partial consequence of the lack of correlation between degeneration and renal cortical echogenicity and the very high variability in the MGV calculated on normal samples (Tables 1 and 2).

MGV was always measured in the most proximal part of the renal cortex and focal hyperechoic areas caused by anisotropic ultrasound backscatter were carefully avoided. It is authors’ opinion that, in the dog, at least part of the MGV variability in the normal samples could be explained by the anisotropic interaction between the ultrasound beam and the renal tubules [7].

The mean age of the dogs included in the present study was relatively high (9.5 ± 4 years) and all the histopathological lesions encountered were related to chronic rather than acute kidney disease; therefore, no data on the relationship between acute renal disease and renal echogenicity was available in this study. Some acute renal pathologies (e.g. ethylene glycol toxicity, leptospirosis) [1] are reported to dramatically increase renal cortical echogenicity; it is very likely that, in dogs, quantitative analysis of ultrasonographic images may be more accurate in the detection of acute rather than chronic renal disease. For this reason further studies, recording also clinical and biochemical data, are wished for the analysis of the relationship occurring between acute kidney disease and renal cortical echogenicity.

The results of this study revealed a significant linear correlation between the MGV calculated on the cat samples and the degeneration scores, whereas no correlation with the inflammation score was evident. Statistical analysis did not show any significant differences in the MGV of normal (grade 0) and mildly degenerated (grade 1 and 2) samples, whereas the MGV calculated on severely degenerated samples was significantly higher. On the other hand, the univariate analysis revealed a moderate influence of histopathological lesions on MGV (44% of MGV variance was explained by the model).

The poor diagnostic performance of MGV in the detection of pathological kidney changes in the cat – albeit higher than in the dog - is, in the authors’ opinion, mostly associated to a large overlap between the MGV calculated in normal and pathological samples (Table 3).

In the cat, proximal tubular lipidosis is reported to increase renal cortical echogenicity in otherwise architecturally normal kidneys [8]. Interestingly, no statistically significant differences in the measured MGV associated with this anatomical variation were apparent in the present study. All the pathological samples in our study population presented a mixture of histological lesions, and no samples classified as “no relevant findings” showed significant proximal tubular lipidosis. Furthermore, no samples were classified as pathological only on the basis of the presence of proximal tubular lipidosis. Our results suggest that other morphological lesions such as fibrosis, tubular vacuolar degeneration, interstitial nephritis, glomerulonephritis rather than proximal tubular lipidosis have a direct influence on the echogenicity of pathological kidneys (Table 5).

Histopathological lesions were related to chronic rather than acute renal damage also in cats. Some forms of acute renal damages (e.g. lily intoxication) [1] are reported to significantly increase renal cortical echogenicity; it is beyond the authors’ knowledge whether it would be possible to discriminate, by means of quantitative analysis of ultrasonographic images, between acute intoxication and severe degeneration.

In human medicine, a low correlation between renal lesions and ultrasonography has been established [9, 10] and the poor diagnostic value of renal cortical echogenicity in the detection of chronic renal disease has been clearly defined [11]. To the best of the authors’ knowledge, this is the first in-vivo report in veterinary medicine to objectively evaluate renal cortical echogenicity in comparison to the detection of chronic renal damage through histopathology. Further studies involving a larger number of subjects are required to establish the relationship occurring between renal cortical echogenicity and acute renal disease in dogs and cats.

## Conclusions

This is the first in-vivo report in veterinary medicine quantifying the changes in renal cortical echogenicity in relation to renal histopathology. In dogs, the high variability of the echogenicity in the normal kidney and the weak influence of pathological changes on renal cortical echogenicity reveal ultrasonography a poor test to discriminate between normal and chronically affected kidneys. In cats, despite the higher influence of renal damage on renal cortical echogenicity, pathological changes are evident only in chronic stages of disease thus limiting the usefulness of ultrasonography in detecting mild renal disease.

## Abbreviations

ANOVA: Analysis of variance
DICOM: Digital imaging and communications in medicine format
MGV: Mean Gray Value
ROC: Receiver operator characteristics curve
RoI: Region of Interest
